# Attitude and communication skills of German medical students

**DOI:** 10.1186/s13104-021-05901-4

**Published:** 2022-01-10

**Authors:** Oana R. Groene, Maren Ehrhardt, Corinna Bergelt

**Affiliations:** 1 Institute of Biochemistry and Molecular Cell Biology, Center for Experimental Medicine, University Medical Center Hamburg-Eppendorf, 52 Martinistraße, 20246 Hamburg, Germany; 2 Institute of General Practice, University Medical Center Hamburg-Eppendorf, Hamburg, Germany; 3 Institute of Medical Psychology, University Medical Center Hamburg-Eppendorf, Hamburg, Germany; 4 Department of Medical Psychology, Greifswald Medical School, Greifswald, Germany

**Keywords:** Communication skills, Attitudes, Patient orientation, Objective Structured Clinical Examination (OSCE), Medical education

## Abstract

**Objective:**

While the development of communication competencies in medical schools plays a pivotal role in the curriculum, studies show that students’ communication skills and patient-centred attitudes may vary based on gender and ethnicity. The goal of this study was to investigate the socio-demographic factors that influence medical students’ communication abilities and, more specifically, to what extent their attitude toward communication skills learning and patient orientation associate with communication abilities. Our population included medical students admitted in 2017. Used tools included a communication score, the patient-provider orientation and communication skills attitudes scales.

**Results:**

Three hundred and sixty-five students participated in the study (56.4% female, 85.2% German native speakers, mean age 24.2 ± 3.5 years). Female and German native speaking students had a better communication skills OSCE performance, were more patient-oriented and had more positive attitudes toward communication skills learning than male and non-native speaking students**.** There was a significant association between gender, native tongue, attitudes towards communication skills learning and communication skills OSCE performance. In conclusion, to support medical students to improve their communication proficiency and attitudes towards the importance of clear communication and patient-oriented care, medical educators should consider teaching and assessment strategies that address socio-cultural aspects of communication.

## Introduction

One of the main roles of a physician articulated in national medical curriculum frameworks of learning objectives is being a communicator [1]. Despite medical educators’ efforts to promote the development and assessment of physicians’ communication competencies, low levels of patient orientation and deficiencies in communication skills amongst medical students still persist at graduation [2].

The main method to assess medical students’ communication skills are the objective structured clinical examinations (OSCEs). Widely used as an outcome measure for academic success in medicine, OSCEs generally assess students’ knowledge and skills in history taking, diagnostic tests interpretation, communication, and physical examinations and were shown to improve students’ communication skills [3]. Given the complexity of the OSCE tasks that involve transfer of knowledge in clinical contexts, experts claim it is difficult to separate the different measured dimensions of competence.

Physicians’ competencies are referred to in curriculum documents as medical knowledge, clinical skills and professional attitudes [4]. Although mentioned as a learning objective and an important aspect to be considered as part of longitudinal curricula, professional attitudes, unlike communication competencies, are not systematically evaluated in OSCE assessments.

Medical students’ declining positive attitudes toward social issues [5] raise concerns about the potential negative effects of attitudes on communication performance. Previous international studies show mixed results concerning the effects of socio-demographic factors such as gender [6, 7], language [8], and ethnicity [9] on patient-centred attitudes or communication skills. Additionally, few studies explore the association between attitudes, socio-demographics and communication skills OSCE performance [10].

The goal of this study was to address this gap and investigate which factors influence medical students’ communication skills OSCE performance and, more specifically, to what extent medical students’ attitudes towards communication skills learning and patient orientation are associated with the communication skills OSCE performance.

## Main text

### Methods

This is an observational study examining communication competencies of medical students at one German medical school. Students had started studying medicine in 2017 and were in their second or third year of study. At the end of the fourth and fifth semester, students take two in person OSCEs containing ten five minutes stations (see Table 1) that test their clinical skills in anaesthesiology, cardiology, and internal medicine amongst others. As part of a national project focused on selection procedures, we developed a communication score based on a composite communication OSCE. We summed up the scores of five in person OSCE stations that included a simulated patient interaction and were exclusively focused on communication skills. OSCE stations assessed risk communication, shared decision-making, basic communication techniques, psychosomatic history taking and generic communication skills in a urology clinical scenario. A more in-depth description of OSCE stations was published elsewhere [11]. Table 1 illustrates which five stations were included in the composite communication OSCE.

**Table 1:** Selection of OSCE stations for the composite communication skills OSCE

OSCE stations	Focus on communication	Simulated patient	5 stations included in the composite communication OSCE
**OSCE I**			
Anaesthesiology			
Risk communication	√	√	√
Cardiology			
Shared decision making	√	√	√
Basic communication techniques	√	√	√
**OSCE II**			
General surgery			
Gastroenterology			
Clinical chemistry			
Psychosocial history taking	√	√	√
Generic communication skills—urology scenario	√	√	√

The instruments used for the OSCE assessment were a combination of validated global rating tools (for generic communication skills and shared decision-making) [12, 13] and checklist instruments that reflected curriculum learning objectives. The scores obtained in the individual OSCE stations (a maximum of 20 points) were added up to form a communication skills score with a maximum of 100 possible points—the main outcome measure of this study.

Socio-demographic data as well as data on attitudes towards communication skills learning and patient orientation were collected as part of a survey conducted electronically at the end of the OSCE.

Patient orientation was measured with the patient-provider orientation scale [14]. The instrument addresses two dimensions and contains twelve 1–6 Likert-type scale items: “sharing” statements refer to the extent to which respondents believe that patients want information and are involved in shared decision-making processes, while “caring” statements reflect the extent to which respondents believe that the expectations, wishes and circumstances of a patient play an important role in the patient’s care and treatment.

Attitudes towards communication skills learning were assessed with the communication skills attitudes scale (CSAS) [15]. The CSAS validated German version of the scale consists of nineteen 1–5 Likert type items of agree-disagree statements about communication skills learning. The instrument has two subscales of negative (NAS, e.g. “I find it difficult to take communication skills learning seriously”) and positive attitudes (PAS, e.g. “learning communication skills helps me understand patients’ rights such as doctor-patient confidentiality and informed consent”), which are calculated as sums.

The data were collected between July 2019 and February 2020.

We report on descriptive analyses of socio-demographic characteristics and communication skills OSCE performance. A one-way between subjects ANOVA/post hoc Bonferroni procedure was conducted to compare the effect of mother tongue on the communication score. We used the T-test to investigate the effect of gender on the communication skills OSCE performance, attitudes towards communication skills learning and patient orientation. Correlation coefficients and backward multiple linear regression models were then calculated to investigate the extent to which gender, mother tongue, attitudes towards communication skills learning and patient orientation are associated with the communication skills score.

### Results

The sample consisted of 365, 2nd or 3rd year medical students who completed all five stations of the composite communication skills OSCE. Students were aged on average 24.8 (± 3.5) years; the majority were female (56.4%, N = 206) and German native speakers (85.2%, N = 311).

There was a significant effect of mother tongue on students’ communication skills OSCE performance, attitudes towards communication skills learning and patient orientation. The communication skills score of native speakers and bilingual medical students was significantly higher than the communication skills score of non-native speakers. German native speakers reported significantly lower NAS and more positive patient oriented attitudes than bilingual or non-native speakers. Female students scored higher on PAS and the communication skills score, lower on NAS and were more patient oriented than male students. Age seemed to play a role in attitudes development in that older students reported more positive and less negative attitudes towards communication skills learning (Table 2).

**Table 2:** Distribution of communication score, attitudes and patient orientation based on socio-demographic characteristics

	Communication score M (SD)	p-value	Positive Attitudes Subscale M (SD)	p-value	Negative Attitudes Subscale M (SD)	p-value	Patient orientation M (SD)	p-value
**Gender**		< 0.001*		0.001*		< 0.001*		< 0.001*
Male (N = 159)	72.9 (7.2)		41.0 (7.1)		16.7 (4.7)		52.1 (6.8)	
Female (N = 206)	77.6 (5.9)		43.3 (6.6)		14.8 (3.6)		54.8 (5.5)	
**Mother tongue**		< 0.001**		0.904		< 0.001**		< 0.001**
German (N = 311)	76.4 (6)		42.3 (6.9)		15.3 (4.0)		54.1 (5.9)	
Bilingual (N = 12)	76.2 (7.9)		43.2 (6.9)		15.2 (3.9)		55.2 (5.5)	
Other (N = 42)	69.4 (9.3)		42.2 (7.1)		18.8 (4.4)		49.6 (7.4)	
**Age**	r − 0.04		r 0.11		r − 0.13		r − 0.02	
		0.481		0.036***		0.011***		0.711

*T-Test

**ANOVA

***Pearson’s correlation

Our regression model explaining 20.7% of the variance revealed that expressing less negative attitudes towards communication skills learning, being a native speaker, and female gender were significantly associated with a higher communication skills score. Positive attitudes, age and patient orientation were excluded from the model due to lack of statistical significance (Table 3).

**Table 3:** Regression model predicting communication skills OSCE performance

	b	Standard Error	p	F	df	R2	R	p
**Initial model**				15.847	6−356	0.211	0.459	<0.001
Negative Attitudes Subscale	− 0.349	0.106	0.001					
Positive Attitudes Subscale	− 0.050	0.058	0.386					
Patient orientation	− 0.025	0.060	0.681					
Mother tongue	− 4.716	1.101	<0.001					
Gender	− 3.538	0.693	<0.001					
Age	− 0.079	0.096	0.413					
**Final model**				31.281	3−359	0.207	0.455	<0.001
Negative Attitudes Subscale	− 0.277	0.084	0.001					
Mother tongue	− 4.928	1.068	<0.001					
Gender	− 3.450	0.680	<0.001					

## Discussion

A composite OSCE focused on communication skills was developed to investigate associations between medical students’ communication skills OSCE performance, attitudes towards communication skills learning, patient orientation and socio-demographic characteristics.

In our study, we found gender differences in the communication skills score in favour of women, a finding which is in line with previous research [9, 16, 17]. Female participants in our sample also expressed more positive attitudes towards communication skills learning and were more patient-oriented than male. Female gender was previously found to be a good predictor of positive attitudes towards patient-centred care [7] and communication skills learning and female doctors are known to spend more time with their patients talking about psychosocial factors that influence health [17]. Gender aspects need to be more thoroughly addressed in the development of the communication skills curriculum to include strategies that specifically support male students in improving their communication proficiency and fostering more positive attitudes towards communication skills learning and patient orientation.

Native speakers as well as bilingual students had significantly better communication skills scores than non-native speaking students. This gap in exam results was also observed in studies from the Netherlands and the UK, which showed that students that belonged to specific ethnic minority groups underperformed in communication and clinical problem-solving tests [9, 18]. Language fluency surely constitutes a strength for native speaking students, especially in the context of exams such as the OSCEs where tasks need to be completed in only five minutes. Non-native speaking students should be provided with additional interactive online courses, extra language classes and tailored assessment methods to adjust for the potential additional cognitive load [19]. Additionally, involving multicultural groups in designing OSCEs should be considered to ensure more universal and culturally appropriate scenarios. Given that diversifying the healthcare force is a societal need, medical education needs to contribute to providing all students with equal opportunities to thrive.

The communication skills score in our study was associated with less NAS, a finding which is congruent with previous research that indicated a link between poorer communication skills and less patient-centred attitudes [10]. Besides language competency, cultural norms, expectations as well as social networks and learning styles could represent an obstacle in students achieving better marks in OSCEs. Although mentioned as an important ingredient to becoming good communicators, professional attitudes seem to remain part of a “hidden curriculum”, with little clarity on teaching and assessment strategies [20].

In conclusion, medical educators need to consider different curriculum design approaches to address attitudes and socio-cultural aspects of communication skills. Such approaches include: supporting peer assisted learning and assessment methods [21] to encourage students to learn from each other and as a vehicle to share experiences and shape attitudes; increasing emphasis on bioethics and intercultural communication teaching; using patients and carers as teachers to improve students’ understanding of patients’ experiences; presenting case studies about different healthcare systems to facilitate a better understanding of how it shapes both patients’ and professionals’ expectations; and the use of simulated patients as trained observers for the assessment of students’ attitudes towards patient orientation.

## Limitations

This study was conducted at one university in Germany. However, research results are coherent with previous international findings and therefore relevant to an international audience of medical educators. This is a cross-sectional study on students’ communication skills in the first half of their studies. A longitudinal study design that allows the observation of attitudes and skills evolution could strengthen claims of decline in communication skills as students progress towards graduation. Finally, we used a validated tool for the self-reporting of attitudes towards communication skills learning. A direct observation of attitudes may have yielded a more robust assessment. Future research should address these gaps as well as the effectiveness of curriculum interventions as proposed above by using both quantitative and qualitative research methods.

## Data Availability

The datasets used and/or analysed during the current study are available from the corresponding author on reasonable request.

## Abbreviations

OSCE: Objective structured clinical examination
CSAS: Communication skills attitudes scale
NAS: Negative Attitudes Subscale
PAS: Positive Attitudes Subscale
